# Supplementary material for: Mass spectrometry uncovers intermediates and off-pathway complexes for SNARE complex assembly
Source: Commun Biol. 2023 Feb 20;6:198. doi: 10.1038/s42003-023-04548-0 (PMC9941103; doi:10.1038/s42003-023-04548-0)
Supplement: Supplementary file 2 — Supplementary Information [file 42003_2023_4548_MOESM2_ESM.pdf]

Supplementary Information

Mass Spectrometry Uncovers Intermediates and Off-pathway Complexes for SNARE Complex Assembly

Julia Hesselbarth[1,#] and Carla Schmidt[1,#]*

[1]Interdisciplinary Research Centre HALOmem, Charles Tanford Protein Centre, Institute of Biochemistry and Biotechnology, Martin Luther University Halle-Wittenberg, Halle, Germany.
#present address: Department of Chemistry – Biochemistry, Biocenter II, Johannes Gutenberg University Mainz, Mainz, Germany.

*Correspondence: carla.schmidt@uni-mainz.de

Figures

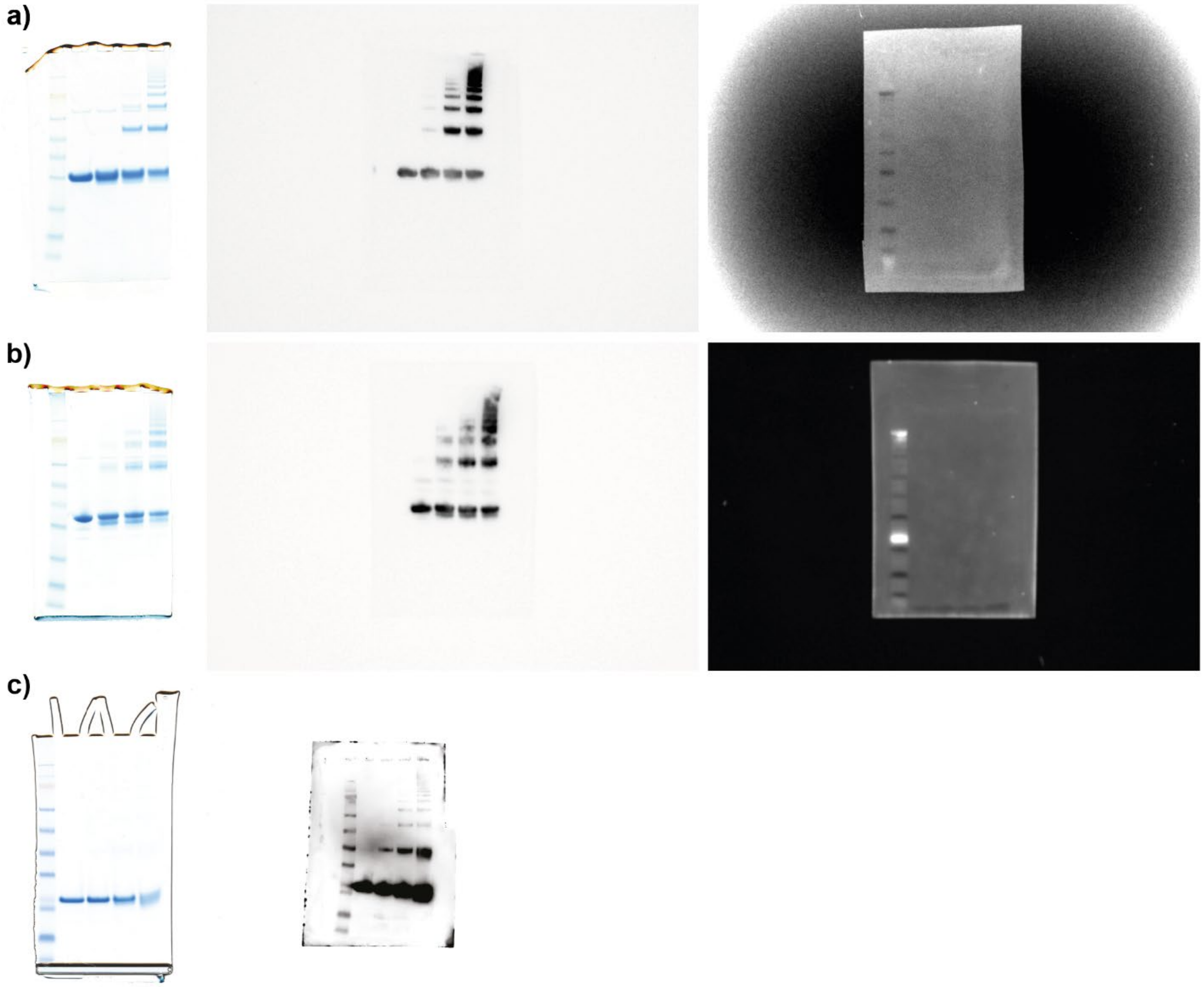

Supplementary Figure 1.

Uncropped gel and western blot images of Figure 1. a) SNAP25. **b)** Syntaxin-1A. **c)** Complexin-1.

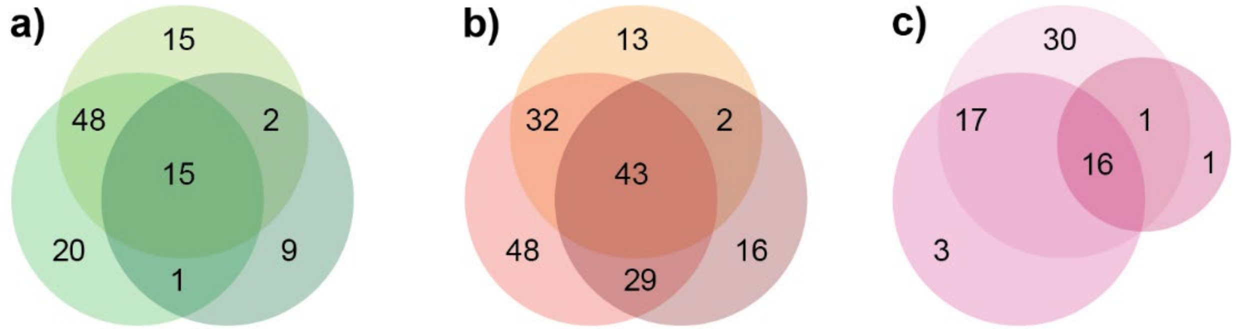

Supplementary Figure 2.

Venn diagrams comparing the number of identified cross-links of individually cross-linked proteins. The number of cross-links after data evaluation is given for three replicates of each protein. **a)** SNAP25. **b)** Syntaxin-1A. **c)** Complexin-1.

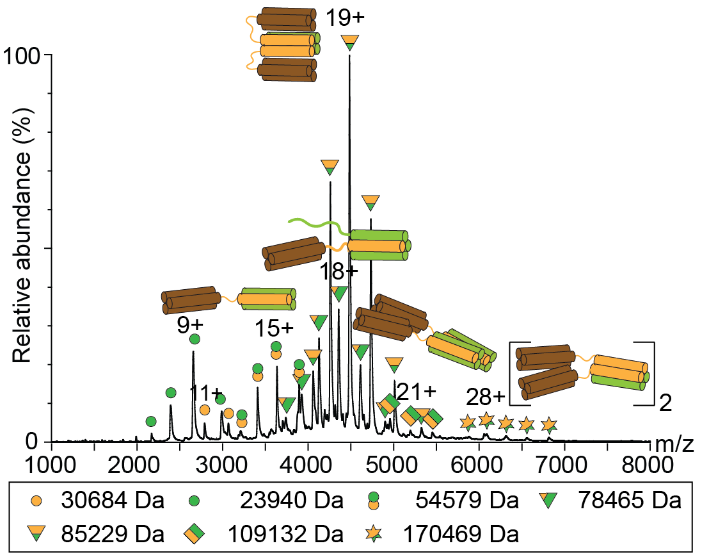

Supplementary Figure 3.

SNAP25:Syntaxin-1A binary complexes. SNAP25 and Syntaxin-1A were incubated at a 1:1 molar ratio and assembled complexes were analysed by native MS. The monomeric proteins (green and yellow circles) as well as SNAP25:Syntaxin-1A complexes with 1:1 (green-yellow twin-circles), 2:1 (green triangle with yellow left corner), 1:2 (yellow triangle with green bottom corner), 2:2 (yellow/green squares) and 2:4 (yellow stars with green corner) stoichiometry were observed. The observed molecular weight is given for each complex (see legend).

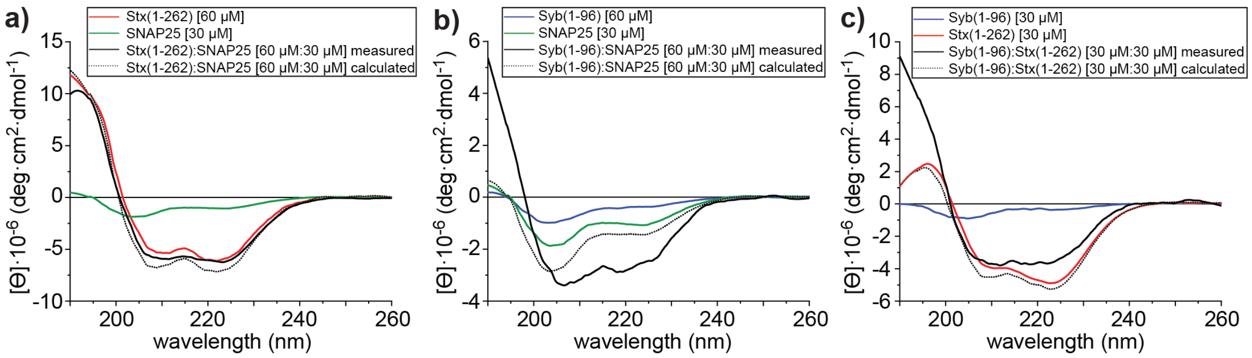

Supplementary Figure 4.

CD spectra comparing monomeric SNARE proteins and binary SNARE complexes. CD spectra of monomeric SNAP25 (green), Syntaxin-1A (1-262) (red) and Synaptobrevin-2 (1-96) (blue) are shown as well as calculated (dotted black lines) and measured (solid black lines) CD spectra of the binary complexes **a**) Syntaxin-1A:SNAP25 2:1 **b**) Synaptobrevin-2:SNAP25 2:1 and **c**) Synaptobrevin-2:Syntaxin-1A 1:1.

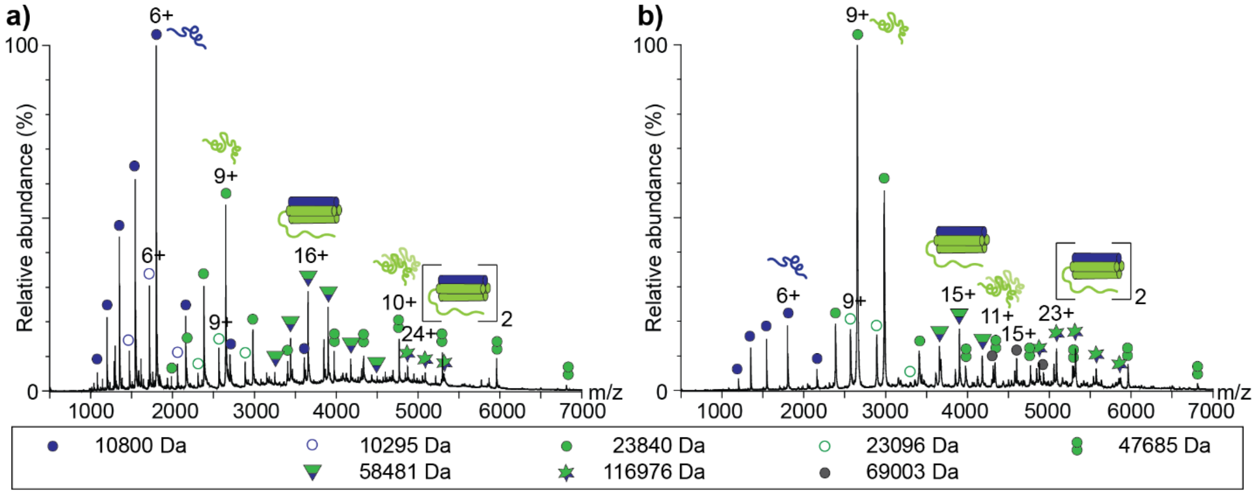

Supplementary Figure 5.

SNAP25:Synaptobrevin-2 binary complexes. SNAP25 and Synaptobrevin-2 were mixed in different ratios. Monomeric SNAP25 (green circles) and Synaptobrevin-2 (blue circles) as well as binary complexes with a stoichiometry of 2:1 (green-blue triangle) and 4:2 (green-blue hexagonal stars) are assigned. Degradation products of SNAP25 and Synaptobrevin-2 (open circles) as well as dimeric SNAP25 (green twin-circles) are also assigned. **a)** SNAP25:Synaptobrevin-2 1:2. **b)** SNAP25:Synaptobrevin-2 2:1. The observed molecular weight is given for the proteins and the complexes (see legend).

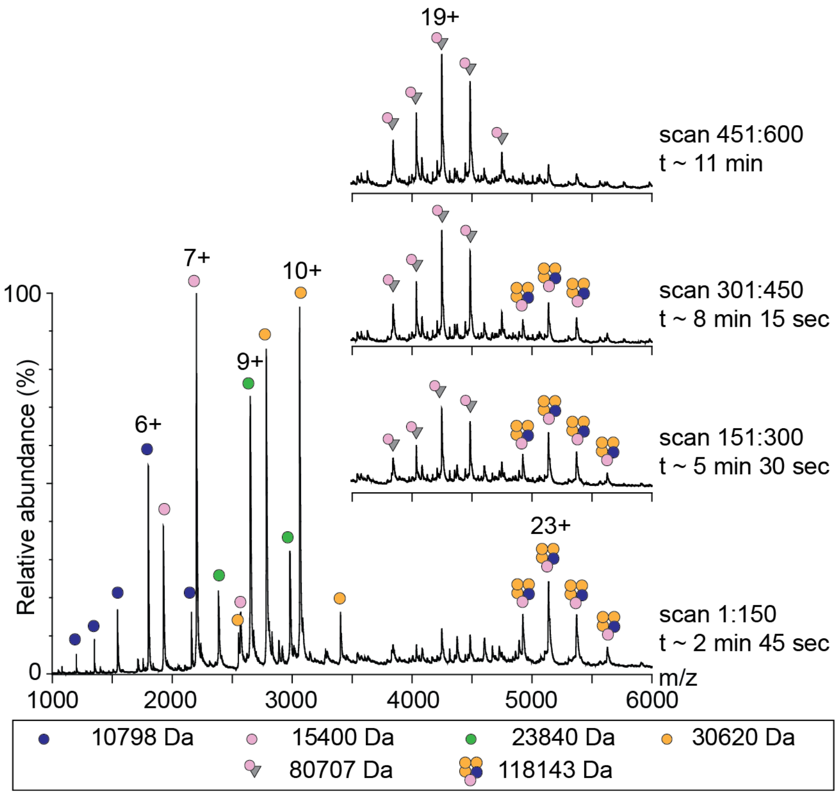

Supplementary Figure 6.

Rearrangement of the ternary Synaptobrevin-2:Syntaxin-1:Complexin-1 complex. Synaptobrevin-2, Syntaxin-1A and Complexin-1 were pre-incubated forming the ternary 1:3:1 complex (main panel, yellow-blue-pink circles). Upon addition of SNAP25, the ternary SNARE complex binding one Complexin-1 molecule is formed (grey triangle-pink circle). Rearrangements of the pre-assembled complex occur over several minutes (scan time 1s, inter scan time 0.1 s); at incubation times above 11 minutes, the SNARE:Complexin-1 complex is exclusively observed. The observed molecular weight is given for the proteins and the complexes (see legend).

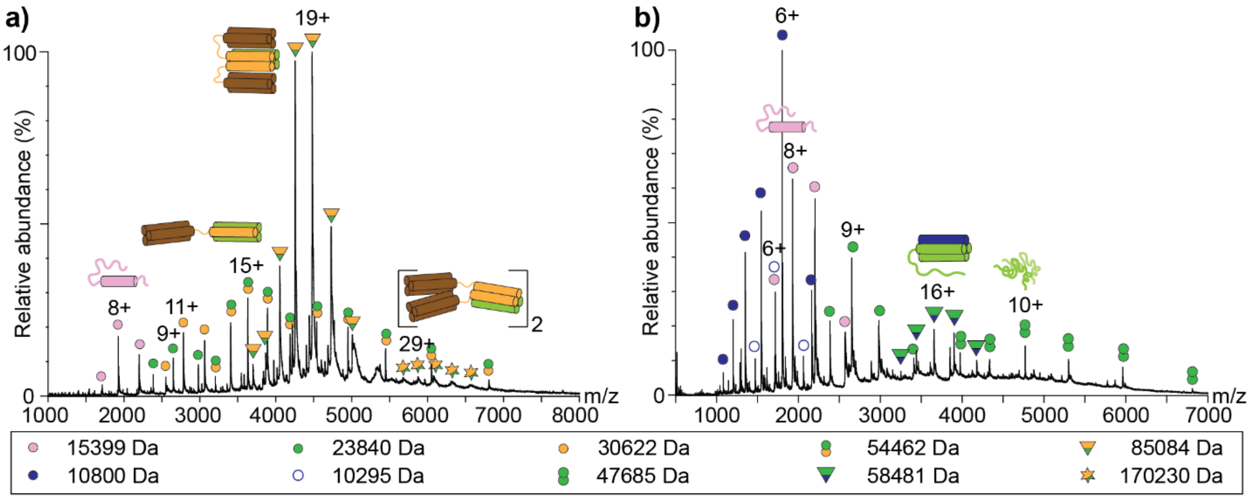

Supplementary Figure 7.

Binary SNARE complexes are not interacting with Complexin-1. Complexin-1 was added to the pre-assembled complexes. Binding of Complexin-1 was not observed. **a)** SNAP25:Syntaxin-1A complex (2:1). **b)** SNAP25:Synaptobrevin-2 complex (2:1). The observed molecular weight is given for the proteins and the complexes (see legend).

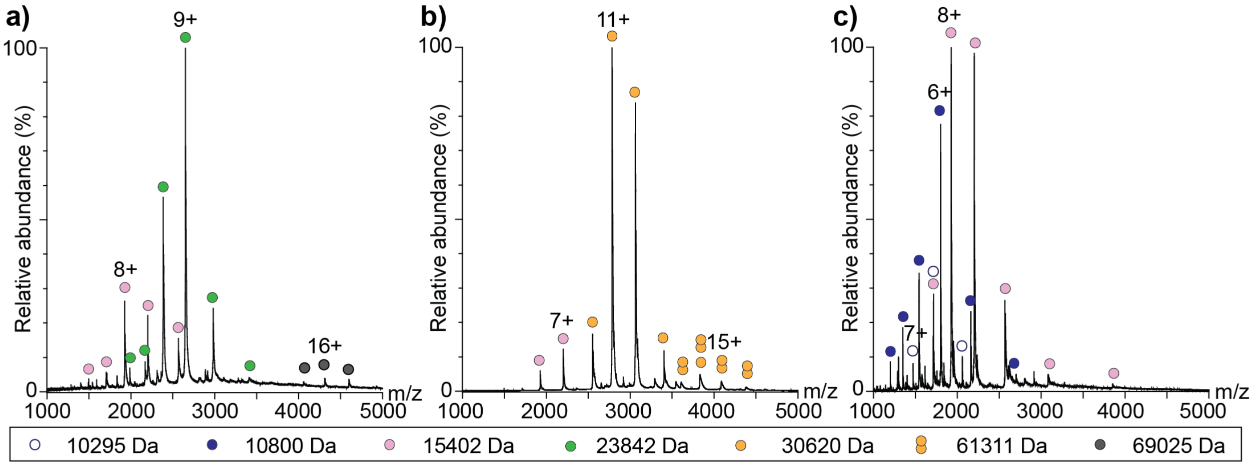

Supplementary Figure 8.

Individual SNARE proteins do not interact with Complexin-1. The SNAREs were incubated with Complexin-1 (pink circles) followed by native MS analysis. Only monomeric proteins were observed. **a)** SNAP 25 (green circles). **b)** Syntaxin-1A (yellow circles). **c)** Synaptobrevin-2 (blue circles). The observed molecular weight is given for all proteins (see legend).

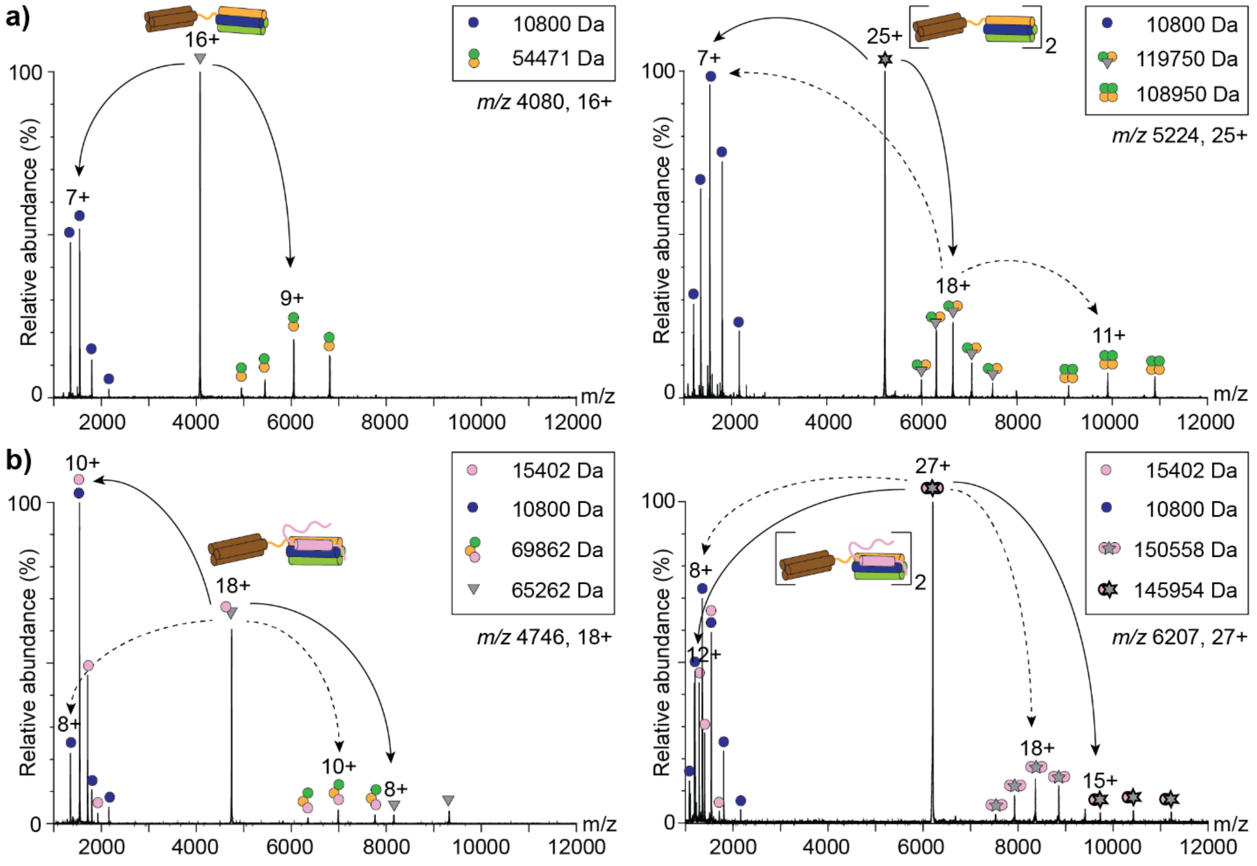

Supplementary Figure 9.

Collision induced dissociation of the ternary SNARE complex and the SNARE:Complexin-1 complex. a) The SNARE complex was formed and the 16+ (lhs) and the 25+ (rhs) charge states of the monomeric and dimeric SNARE complexes, respectively, were selected for collisional induced dissociation. Synaptobrevin-2 dissociates from both complexes yielding the highly charged monomeric protein and a stripped complex omitting Synaptobrevin-2. Note that for the dimeric SNARE complex two consecutive dissociation events occurred yielding first and second generation dissociation products omitting one or two Synaptobrevin-2 molecules. **b)** The 18+ (lhs) and the 27+ (rhs) charge states of the monomeric or dimeric SNARE:Complexin-1 complexes, respectively, were selected for collision induced dissociation. Two dissociation events occurred; dissociation of highly charged monomeric Synaptobrevin-2 and Complexin-1, respectively, generated stripped complexes omitting either of the two subunits. The observed molecular weight is given for the proteins and the complexes (see legend).

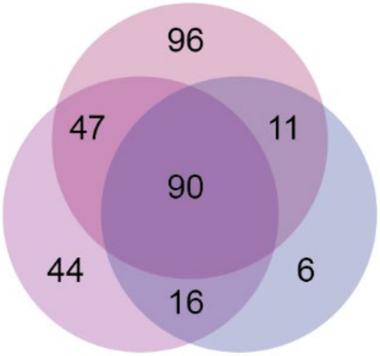

Supplementary Figure 10.

Venn diagram comparing the number of identified cross-links within the SNARE:Complexin-1 complex. The SNARE:Complexin-1 complex was cross-linked with BS3. The number of identified cross-links after data evaluation is given for each of three replicates. 90 cross-links were identified in all three replicates. 164 cross-links were identified in two out of three replicates.

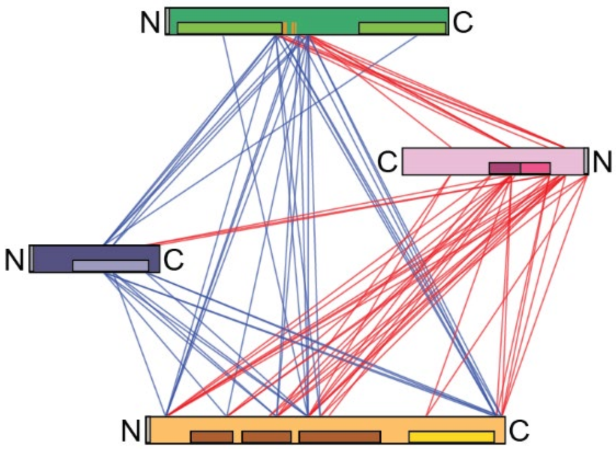

Supplementary Figure 11.

Network plot visualising protein interactions within the SNARE:Complexin-1 complex. Proteins are shown as coloured bars (SNAP25, green; Synaptobrevin-2, blue; Syntaxin-1A, orange; Complexin-1, pink). The length of the bars corresponds to the protein length. N- and C-termini are indicated. SNARE motifs, alpha-helices of the Habc domain of Syntaxin-1A and the central and accessory helices of Complexin-1 are indicated (green, blue, yellow, brown, purple, pink boxes). Mutated cysteine residues in SNAP25 are shown (orange lines). Solid lines represent cross-links identified in at least two replicates. Interactions between SNARE proteins (blue) and between Complexin-1 and the SNARE proteins (red) are shown.

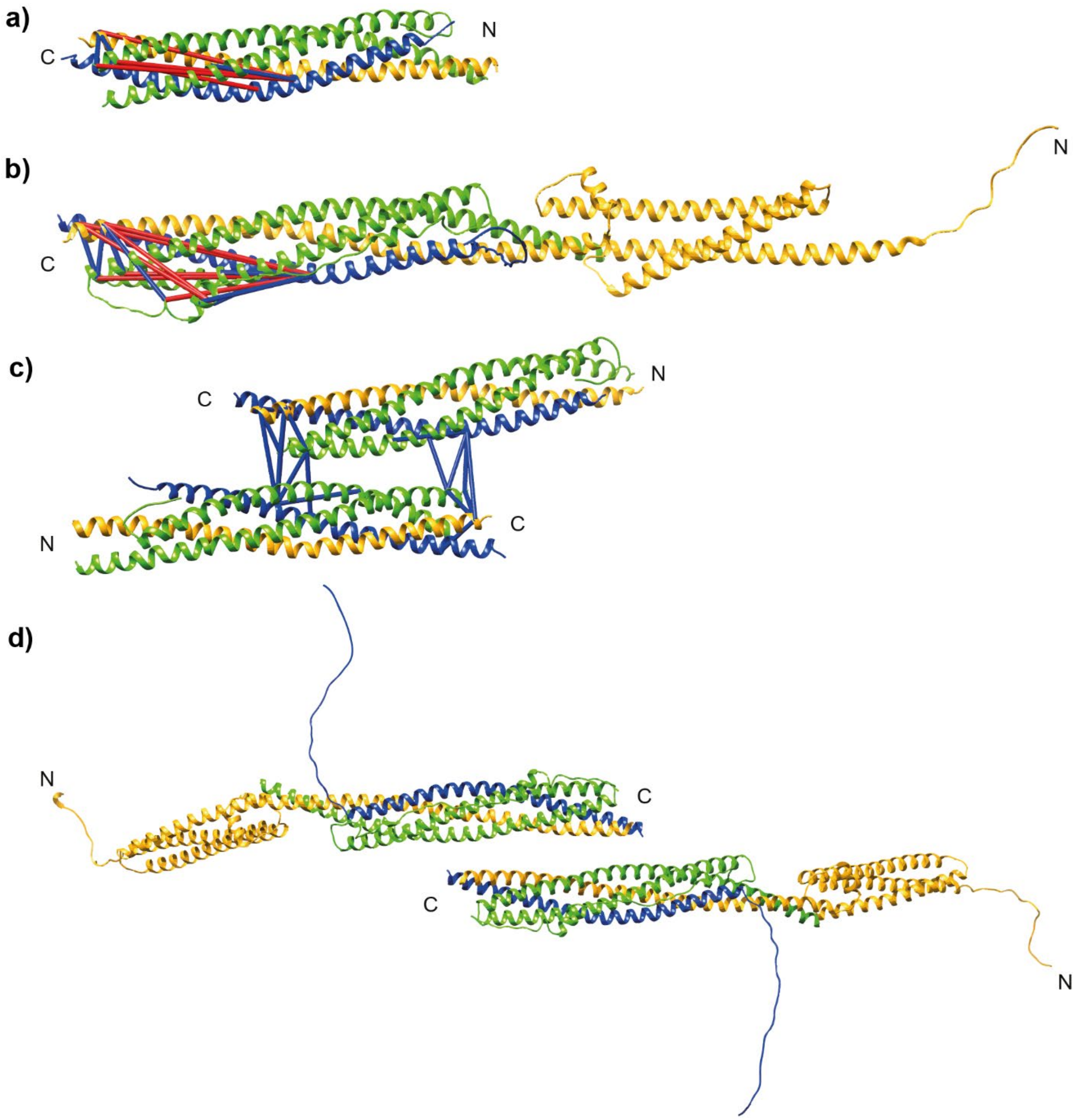

Supplementary Figure 12.

Validation of inter-molecular interactions between SNARE proteins. Proteins are shown in cartoon representation (SNAP25, green; Synaptobrevin-2, blue; Syntaxin-1A, orange). N- and C-termini are indicated. Short-range (<30 Å, blue lines) and long-range (>30 Å, red lines) cross-links identified in at least two of three replicates are visualised. **a)** 8 of 16 cross-links could be mapped onto a high-resolution structure of the SNARE complex (PDB-ID: 1SFC). Flexible regions of the proteins are not included, and cross-links identified in these regions are, therefore, not shown. **b**) All 16 cross-links between SNARE proteins are visualised in an AlphaFold2-multimer prediction of the SNARE:Complexin-1 complex including flexible regions. 7 long-range and 9 short-range cross-links were observed. **c)** 8 of 16 cross-links are visualised in two antiparallel-oriented high-resolution structures of SNARE complexes. All cross-linking distances are satisfied. Note that cross-links are shown twice. **d)** A dimer of the SNARE complex including flexible regions and the Habc domain of Syntaxin-1A predicted by AlphaFold2-multimer confirms an antiparallel orientation of the SNARE complexes.

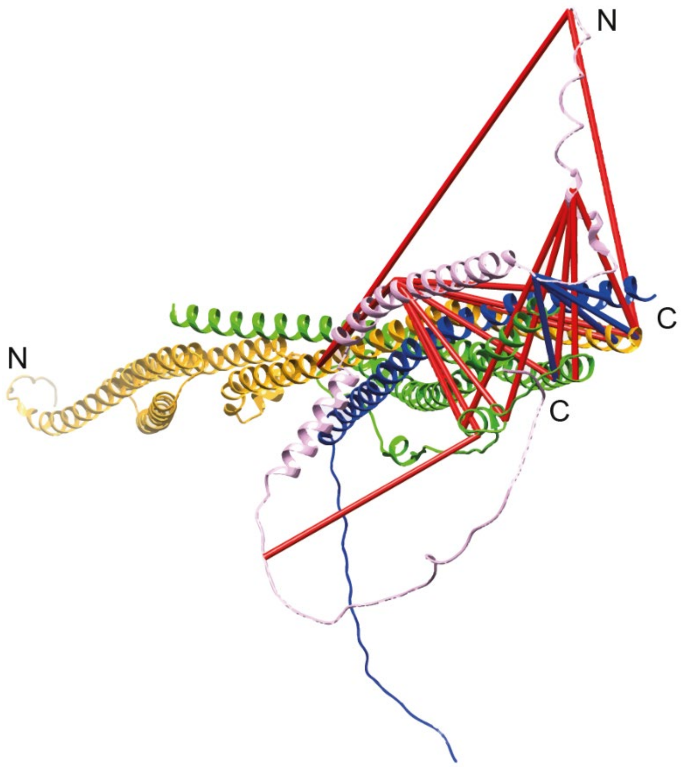

Supplementary Figure 13.

Visualisation of inter-molecular interactions of Complexin-1 within the SNARE:Complexin-1 complex. Proteins are shown in cartoon representation (SNAP25, green; Synaptobrevin-2, blue; Syntaxin-1A, orange; Complexin-1, pink). N- and C-termini are indicated. Cross-links (red and blue lines), which were identified in at least two of three replicates, are visualised in an AlphaFold2-multimer prediction of the SNARE:Complexin-1 complex, including flexible regions of the proteins. Note that flexible regions are captured in one conformation rather than multiple possible conformations. 21 of the 37 cross-links formed in the SNARE:Complexin-1 complex are visualised. 16 long-range cross-links (>30 Å, red lines) were observed involving flexible regions of SNAP25 and Complexin-1. 5 short-range cross-links were observed between the N-terminal domain of Complexin-1 and the C-terminal SNARE complex.

Tables

Supplementary Table 1.

Protein identification. The hit number, the protein name (and database accession number of contaminating *E. coli* proteins), the number of identified peptides, the observed sequence coverage and the MaxQuant (MQ) protein score are given for each protein purified in the different samples.

Protein sample	Hit	Protein	Peptides	Sequence coverage [%]	MQ Score
Complexin-1	1	Complexin-1	19	88.3	323.31
	2	Serine hydroxymethyltransferase (A0A140N8X9)	6	19.9	86.90
	3	Histidine triad (HIT) protein (A0A140ND09)	7	96.6	83.33
	4	Dihydrolipoyl dehydrogenase (A0A140NBS3)	6	15.0	44.00
	5	ATP synthase subunit alpha (A0A140ND72)	4	9.6	30.45
SNAP25 (CtoS)	1	SNAP25 (CtoS)	38	94.7	323.31
	2	Chaperone protein DnaK (A0A140NFV3)	46	74.5	323.31
	3	Elongation factor Tu (A0A140NCI6;A0A140N6W0)	15	52.8	130.83
	4	ATP synthase subunit beta (A0A140NHS0)	13	38.0	107.06
	5	Dihydrolipoyl dehydrogenase (A0A140NBS3)	12	34.2	106.18
Syntaxin (1-262)	1	Syntaxin(1-262)	33	83.8	323.31
	2	Chaperone protein DnaK (A0A140NFV3)	32	57.8	267.96
	3	SNAP25(CtoS)	18	70.8	224.46
	4	ATP synthase subunit beta (A0A140NHS0)	10	29.3	74.34
	5	30S ribosomal protein S1 (A0A140NBA5)	8	18.5	54.46
Synaptobrevin (1-96)	1	Synaptobrevin (1-96)	16	94.9	323.31
	2	Ferric uptake regulation protein (A0A140NE13)	8	65.5	117.29
	3	PTS system, mannose/fructose/sorbose family, IIA subunit (A0A140NBF2)	6	24.5	49.61
	4	SNAP25 (CtoS)	4	26.3	37.39
	5	Outer membrane chaperone Skp (OmpH) (A0A140NFE9)	2	14.9	31.55

Supplementary Table 2.

Masses of protein and protein complexes determined by native MS experiments. The figure number, the protein and protein complexes, the theoretically calculated masses based on the amino acid sequences and the experimentally determined masses including standard deviations are given.

Figure number	Protein (complexes)	Theoretical mass (Da)	Experimental mass (Da)
1	SNAP25	23553.13	23926 ± 5.04
	SNAP25 dimer	47106.26	47841 ± 5.04
	SNAP25 trimer	70659.39	71754 ± 8.32
	Stx(1-262)	30620.21	30623 ± 3.52
	Stx(1-262) dimer	61240.42	61366 ± 34.94
	Stx(1-262) trimer	91860.63	92009 ± 92.36
	Cpx1	15403.37	15403 ± 0.34
	Cpx1 dimer	30806.74	30807 ± 1.05
	Cpx1 trimer	46210.11	46284 ± 11.34
2	SNAP25	23553.13	23840 ± 0.58
	Stx(1-262)	30620.21	30622 ± 2.30
	Syb(1-96)	10799.23	10799 ± 0.24
	SNAP25:Stx(1-262) 1:1	54173.34	54464 ± 5.00
	SNAP25:Stx(1-262) 1:2	84793.55	85091 ± 18.49
	SNAP25:Stx(1-262) 2:4	169587.10	170278 ± 18.56
	SNAP25:Stx(1-262):Syb(1-96) 1:1:1	64972.57	65308 ± 6.82
	SNAP25:Stx(1-262):Syb(1-96) 2:2:2	129945.14	130603 ± 11.23
	SNAP25:Stx(1-262):Syb(1-96) 3:3:3	194917.71	195970 ± 14.67
3	SNAP25	23553.13	23840 ± 0.58
			23096 ± 3.05
	SNAP25 dimer	47106.26	47694 ± 12.67
	Stx(1-262)	30620.21	30622 ± 2.30
	Syb(1-96)	10799.23	10800 ± 0.58
			10295 ± 0.99
	DnaK	69115.00	69029 ± 10.00
	SNAP25:Syb(1-96) 2:1	57905.49	58513 ± 1.53
	SNAP25:Syb(1-96) 4:2	115810.98	117040 ± 14.46
	SNAP25:Stx(1-262):Syb(1-96) 1:1:1	64972.57	65338 ± 10.06
	SNAP25:Stx(1-262):Syb(1-96) 2:2:2	129945.14	130659 ± 13.66
	SNAP25:Stx(1-262):Syb(1-96) 3:3:3	194917.71	196053 ± 46.80
	SNAP25:Stx(1-262):Syb(1-96) 4:4:4	259890.28	261438 ± 95.99

	SNAP25	23553.13	23840 ± 0.58
	Stx(1-262)	30620.21	30622 ± 2.30
	Stx(1-262) dimer	61240.42	61246 ± 2.52
	Syb(1-96)	10799.23	10800 ± 0.57
	Cpx1	15403.37	15400 ± 1.16
4	Stx(1-262):Cpx1 3:1	107264.00	107276 ± 11.79
	Stx(1-262):Syb(1-96) 3:1	102659.86	102661 ± 15.62
	Stx(1-262):Syb(1-96):Cpx1 3:1:1	118063.23	118181 ± 7.22
	SNAP25:Stx(1-262):Syb(1-96):Cpx1 1:1:1:1	80375.94	80695 ± 3.78
	SNAP25:Stx(1-262):Syb(1-96):Cpx1 1:4:2:2	198439.17	198997 ± 12.82
	SNAP25	23553.13	23848 ± 0.99
	Stx(1-262)	30620.21	30623 ± 3.52
	Syb(1-96)	10799.23	10800 ± 0.58
	Cpx1	15403.37	15400 ± 1.16
	DnaK	69115.00	69014 ± 6.03
5	SNAP25:Stx(1-262):Syb(1-96) 1:1:1	64972.57	65306 ± 8.15
	SNAP25:Stx(1-262):Syb(1-96) 2:2:2	129945.14	130632 ± 7.51
	SNAP25:Stx(1-262):Syb(1-96) 3:3:3	194917.71	195970 ± 25.36
	SNAP25:Stx(1-262):Syb(1-96):Cpx1 1:1:1:1	80375.94	80713 ± 2.32
	SNAP25:Stx(1-262):Syb(1-96):Cpx1 2:2:2:2	160751.88	161437 ± 20.41
	SNAP25	23553.13	23940 ± 9.11
	Stx(1-262)	30620.21	30694 ± 10.65
	SNAP25:Stx(1-262) 1:1	54173.34	54579 ± 21.40
S3	SNAP25:Stx(1-262) 2:1	77726.47	78465 ± 7.86
	SNAP25:Stx(1-262) 1:2	84793.55	85229 ± 5.61
	SNAP25:Stx(1-262) 2:2	108346.68	109132 ± 11.32
	SNAP25:Stx(1-262) 2:4	169587.10	170469 ± 41.43
	Syb(1-96)	10799.23	10800 ± 0.57
			10295 ± 0.99
S5	SNAP25	23553.13	23840 ± 0.58
			23096 ± 3.05
	SNAP25 dimer	47106.26	47685 ± 4.04
	SNAP25:Syb(1-96) 2:1	57905.49	58481 ± 2.89
	SNAP25:Syb(1-96) 4:2	115810.98	116976 ± 1.54
	DnaK	69115.00	69003 ± 14.24

S6	Cpx1	15403.37	15400 ± 1.16
	SNAP25	23553.13	23840 ± 0.57
	Stx(1-262)	30620.21	30620 ± 1.57
	Syb(1-96)	10799.23	10798 ± 0.14
	SNAP25:Stx(1-262):Syb(1-96):Cpx1 1:1:1:1	80375.94	80707 ± 6.93
	Stx(1-262):Syb(1-96):Cpx1 3:1:1	118063.23	118143 ± 7.51
S7	Cpx1	15403.37	15399 ± 0.58
	SNAP25	23553.13	23840 ± 0.58
	SNAP25 dimer	47106.26	47685 ± 4.03
	Stx(1-262)	30620.21	30622 ± 2.30
	Syb(1-96)	10799.23	10800 ± 0.57
			10295 ± 0.99
	SNAP25:Stx(1-262) 1:1	54173.34	54462 ± 2.64
	SNAP25:Stx(1-262) 1:2	84793.55	85084 ± 6.42
	SNAP25:Stx(1-262) 2:4	169587.1	170230 ± 19.00
	SNAP25:Syb(1-96) 2:1	57905.49	58481 ± 2.89
S8	Cpx1	15403.37	15402 ± 0.58
	SNAP25	23553.13	23842 ± 0.57
	DnaK	69115.00	69025 ± 5.51
	Stx(1-262)	30620.21	30620 ± 0.52
	Stx(1-262) dimer	61240.42	61311 ± 6.99
	Syb(1-96)	10799.23	10295 ± 0.99
			10800 ± 0.57
S9	Cpx1	15403.37	15402 ± 4.61
	Syb(1-96)	10799.23	10800 ± 0.57
	SNAP25:Stx(1-262) 1:1	54173.34	54471 ± 6.43
	SNAP25:Stx(1-262):Syb(1-96) 1:1:1	64972.57	65262 ± 2.11
	SNAP25:Stx(1-262):Cpx1 1:1:1	69576.71	69862 ± 10.79
	SNAP25:Stx(1-262) 2:2	108346.68	108950 ± 8.33
	SNAP25:Stx(1-262):Syb(1-96) 2:2:1	119145.91	119750 ± 9.07
	SNAP25:Stx(1-262):Syb(1-96):Cpx1 2:2:1:2	149952.65	150558 ± 8.89
	SNAP25:Stx(1-262):Syb(1-96):Cpx1 2:2:2:1	145348.51	145954 ± 16.74
