# Supplementary material for: Mass spectrometry uncovers intermediates and off-pathway complexes for SNARE complex assembly
Source: Commun Biol. 2023 Feb 20;6:198. doi: 10.1038/s42003-023-04548-0 (PMC9941103; doi:10.1038/s42003-023-04548-0)
Supplement: Supplementary file 3 — Description of Additional Supplementary Files [file 42003_2023_4548_MOESM3_ESM.pdf]

Description of Additional Supplementary Files

File name: Supplementary Data 1

Description: Cross-links identified in SNAP25(CtoS). Data is shown in Figure 1 and Supplementary Figure 2.

File name: Supplementary Data 2

Description: Cross-links identified in Syntaxin1(1-262). Data is shown in Figure 1 and Supplementary Figure 2.

File name: Supplementary Data 3

Description: Cross-links identified in Complexin-1. Data is shown in Figure 1 and Supplementary Figure 2.

File name: Supplementary Data 4

Description: Cross-links identified in the SNARE:Complexin-1 complex. Data is shown in Figure 6 and Supplementary Figures 10, 11, 12 and 13
