# Supplementary material for: Mass spectrometry uncovers intermediates and off-pathway complexes for SNARE complex assembly
Source: Commun Biol. 2023 Feb 20;6:198. doi: 10.1038/s42003-023-04548-0 (PMC9941103; doi:10.1038/s42003-023-04548-0)
Supplement: Supplementary file 8 — Reporting summary [file 42003_2023_4548_MOESM8_ESM.pdf]

nature portfolio

Corresponding author(s): Carla Schmidt

Last updated by author(s): Jan 24, 2023

Reporting Summary

Nature Portfolio wishes to improve the reproducibility of the work that we publish. This form provides structure for consistency and transparency in reporting. For further information on Nature Portfolio policies, see our Editorial Policies and the Editorial Policy Checklist.

Statistics

For all statistical analyses, confirm that the following items are present in the figure legend, table legend, main text, or Methods section.

n/a	Confirmed	
☐	☒	The exact sample size (*n*) for each experimental group/condition, given as a discrete number and unit of measurement
☐	☒	A statement on whether measurements were taken from distinct samples or whether the same sample was measured repeatedly
☒	☐	The statistical test(s) used AND whether they are one- or two-sided<br>*Only common tests should be described solely by name; describe more complex techniques in the Methods section.*
☒	☐	A description of all covariates tested
☒	☐	A description of any assumptions or corrections, such as tests of normality and adjustment for multiple comparisons
☒	☐	A full description of the statistical parameters including central tendency (e.g. means) or other basic estimates (e.g. regression coefficient) AND variation (e.g. standard deviation) or associated estimates of uncertainty (e.g. confidence intervals)
☒	☐	For null hypothesis testing, the test statistic (e.g. *F*, *t*, *r*) with confidence intervals, effect sizes, degrees of freedom and *P* value noted<br>*Give P values as exact values whenever suitable.*
☒	☐	For Bayesian analysis, information on the choice of priors and Markov chain Monte Carlo settings
☒	☐	For hierarchical and complex designs, identification of the appropriate level for tests and full reporting of outcomes
☒	☐	Estimates of effect sizes (e.g. Cohen's *d*, Pearson's *r*), indicating how they were calculated

Our web collection on statistics for biologists contains articles on many of the points above.

Software and code

Policy information about availability of computer code

Data collection	vendor-specific software for MS instrumentation used (XCalibur v.4.2.47 (Thermo Scientific), MassLynx v.4.1 (Waters))
Data analysis	Open source software for data analysis: MaxQuant v.1.6.17 for protein identification (Cox 2008), pLink v.2.3.9 for cross-linking analysis (Yang 2012), Massign v.11142014 for analysis of native mass spectrometry data (Morgner, Robinson 2012); in-house prepared code for conversion of cross-linking data (CroCo v.0.7.1: Bender, Schmidt 2020); open source software for visualisation of cross-links: XVis web server (Grimm et al 2015), Xlink Analyzer v.1.1.4 (Kosinski et al 2015), Chimera v.1.14 (Petterson et al 2004); open source software for visualisation of crystall structures: PyMOL v1.8 (Schrödinger 2015), AlphaFold (Jumper et al 2021), ColabFold (Mirdita et al 2022)

For manuscripts utilizing custom algorithms or software that are central to the research but not yet described in published literature, software must be made available to editors and reviewers. We strongly encourage code deposition in a community repository (e.g. GitHub). See the Nature Portfolio guidelines for submitting code & software for further information.

Data

Policy information about availability of data

All manuscripts must include a data availability statement. This statement should provide the following information, where applicable:

- Accession codes, unique identifiers, or web links for publicly available datasets
- A description of any restrictions on data availability
- For clinical datasets or third party data, please ensure that the statement adheres to our policy

All MS raw files and the corresponding results files including databases were deposited to the ProteomeXchange Consortium (www.proteomexchange.org) via the PRIDE partner repository with the dataset identifier PXD030619 [https://www.ebi.ac.uk/pride/archive/projects/ PXD030619].

Human research participants

Policy information about studies involving human research participants and Sex and Gender in Research.

Reporting on sex and gender	n/a
Population characteristics	n/a
Recruitment	n/a
Ethics oversight	n/a

Note that full information on the approval of the study protocol must also be provided in the manuscript.

Field-specific reporting

Please select the one below that is the best fit for your research. If you are not sure, read the appropriate sections before making your selection.

☒ Life sciences ☐ Behavioural & social sciences ☐ Ecological, evolutionary & environmental sciences

For a reference copy of the document with all sections, see nature.com/documents/nr-reporting-summary-flat.pdf

Life sciences study design

All studies must disclose on these points even when the disclosure is negative.

Sample size	No statistial method was used for sample size calculation. All proteins were prepared in house. For all cross-linking and native mass spectrometry experiments at least three biological replicates were performed. For protein identification one experiment was performed to verify the protein of interest. An FDR was applied during database search for protein identification and cross-link analysis. Identified cross-links were manually validated. CD spectroscopy was performed at least twice for each sample.
Data exclusions	No data excluded.
Replication	All replicates are biological replicates. Each cross-linking experiment was performed three times. Each native mass spectrometry measurement was performed at least three times. A representative spectrum is shown for each experiment. All attemps at replication were successful. CD measurements were performed at least twice.
Randomization	Randomization is not relevant to the study because only one sample per experiment was used.
Blinding	Blinding was not relevant to the study because the samples are known and no subjective qualitative results were reported.

Reporting for specific materials, systems and methods

We require information from authors about some types of materials, experimental systems and methods used in many studies. Here, indicate whether each material, system or method listed is relevant to your study. If you are not sure if a list item applies to your research, read the appropriate section before selecting a response.

Materials & experimental systems

n/a	Involved in the study
☐	☒ Antibodies
☒	☐ Eukaryotic cell lines
☒	☐ Palaeontology and archaeology
☒	☐ Animals and other organisms
☒	☐ Clinical data
☒	☐ Dual use research of concern

Methods

n/a	Involved in the study
☒	☐ ChIP-seq
☒	☐ Flow cytometry
☒	☐ MRI-based neuroimaging

Antibodies

Antibodies used

anti-SNAP25, anti-Syntaxin-1A and anti-Complexin antibodies obtained from Synaptic Systems GmbH: anti-SNAP25 clone 71.1 (Cat.No. 111 011BT), anti-Syntaxin-1A clone 78.3 (Cat.No. 110 111) and anti-Complexin-1/2 (Cat.No. 122 003).
Secondary antibodies obtainedfrom Sigma-Aldrich: anti-Mouse IgG (whole molecule)–Peroxidase antibody produced in rabbit (Cat.No. A9044), anti-Rabbit IgG (whole molecule)–Peroxidase antibody produced in goat (Cat.No. A9169)

Validation

anti-SNAP25 clone 71.1: validated for human, mouse, rat, vertebrates, invertebrates and for applications of WB, IP, ICC, IHC, IHC-P/FFPE
anti-Syntaxin-1A clone 78.3: validated for human, mouse, rat, mammals, chicken and for applications of WB, IP, ICC, IHC, IHC-P/FFPE, and EM
anti-Complexin-1/2: validated for human, mouse, rat, cow, electric ray, rabbit and for applications of WB, IP, ICC, IHC, IHC-P/FFPE
Anti-Mouse IgG (whole molecule)–Peroxidase antibody produced in rabbit: validated for mouse for applications of WB, IHC and ELISA
Anti-Rabbit IgG (whole molecule)–Peroxidase antibody produced in goat: validated for rabbit for applications of WB, IHC and ELISA
